# Effect of Antiplatelet Therapy on Acute Respiratory Distress Syndrome and Mortality in Critically Ill Patients: A Meta-Analysis

**DOI:** 10.1371/journal.pone.0154754

**Published:** 2016-05-16

**Authors:** Lijun Wang, Heng Li, Xiaofei Gu, Zhen Wang, Su Liu, Liyong Chen

**Affiliations:** Department of Anesthesiology, Daping Hospital, Third Military Medical University, Chongqing, China; King Abdullah International Medical Research Center, SAUDI ARABIA

## Abstract

**Background:**

Antiplatelet agents are commonly used for cardiovascular diseases, but their pleiotropic effects in critically ill patients are controversial. We therefore performed a meta-analysis of cohort studies to investigate the effect of antiplatelet therapy in the critically ill.

**Methods:**

Nine cohort studies, retrieved from PubMed and Embase before November 2015, involving 14,612 critically ill patients and 4765 cases of antiplatelet users, were meta-analysed. The main outcome was hospital or 30-day mortality. Secondary outcome was acute respiratory distress syndrome (ARDS) or acute lung injury (ALI). Random- or fixed-effect models were taken for quantitative synthesis of the data.

**Results:**

Antiplatelet therapy was associated with decreased mortality (odds ratio (OR) 0.61; 95% confidence interval (CI), 0.52–0.71; I^2^ = 0%; *P* <0. 001) and ARDS/ALI (OR 0.64; 95% CI, 0.50–0.82; I^2^ = 0%; *P* <0. 001). In every stratum of subgroups, similar findings on mortality reduction were consistently observed in critically ill patients.

**Conclusions:**

Antiplatelet therapy is associated with reduced mortality and lower incidence of ARDS/ALI in critically ill patients, particularly those with predisposing conditions such as high-risk surgery, trauma, pneumonia, and sepsis. However, it remains unclear whether similar findings can be observed in the unselected and broad population with critical illness.

## Introduction

Sepsis and acute respiratory distress syndrome (ARDS) in the United States both contribute signi-ficantly to the expanding burden of critical illness. The incidence rate of sepsis or septic shock appears to be increasing [1], and with an annual cost of $17 billion, the mortality is reported at approximately 30% in sepsis [2]. Meanwhile, epidemiologic evidences suggest an incidence of 190,600 patients annually for ARDS and the mortality is presently estimated at 25–38% [3–4]. Importantly, sepsis and multiple organ failure (MOF) are leading causes of death in the critically ill. However, among several factors associated with MOF, ARDS, and sepsis, platelet activation and sequestration may play one of the most important roles in micro-vascular thrombosis and releasing of inflammatory mediators through platelet–endothelial interaction [5–7]. Moreover, a decline in circulating platelet count can be commonly observed in critically ill patients, and thrombocytopenia is a powerful predictor of mortality [8–10]. Given these reasons, beneficial effect of impeding platelet activation has been hypothesized for critically ill patients.

Antiplatelet agents are used to reduce platelet activation and their cardiovascular benefits in patients with the risk of heart disease, stroke, and peri-pheral arterial thrombosis are widely accepted in clinical practice. However, their pleiotropic effects in critically ill patients yielded controversial results. Many observational studies have demonstrated a protective effect of antiplatelet agents in critically ill patients [11–13], but some are not [14–15]. More unfortunately, no proper reports exploring the therapeutic effects of antiplatelet agents in randomized controlled studies had been previously published; therefore, we may not definitely know whether antiplatelet agents have beneficial effects, or whether these findings were observed owing to the bias effect.

Up to now, no syntheses of the evidence have been published to recapitulate and summarize the affect of this therapy in ICU population. Consequently, we conducted this meta-analysis to pool the available data and to explore the possible impact of anti-platelet agents on ARDS/ALI and risk of mortality in critically ill patients.

## Methods

This meta-analysis was performed in adherence to the Preferred Reporting Items for Systematic Reviews and Meta-Analyses (PRISMA) [16]. Neither ethical approval nor patient consent was required because this is a meta-analysis on base of previously published studies.

### Search Strategy

Without language restrictions, electronic databases including Pubmed, Embase were retrieved to identify relevant studies from inception to November 10, 2015. A combination of exploded Medical Subject Headings (MESH) terms and corresponding keywords was used while searching. The search terms were as follows: (‘platelet aggregation inhibitor’, ‘antiplatelet’, ‘anti-platelet’, ‘platelet inhibitor’, ‘aspirin’, ‘acetyl salicylic acid’, ‘clopidogrel’) and (‘ARDS’, ‘acute respiratory distress syndrome’, ‘ALI’, ‘acute lung injury’, ‘acute respiratory failure’). Additionally, We hand-searched the reference lists of each selected study, as well as previous review articles to obtain other potential relevant articles.

### Inclusion Criteria

Published cohort studies meeting the following criteria were included: (1) population: critically ill patients, or rather, it referred to ICU or emergency patients with ARDS/ALI predisposing conditions, such as sepsis, septic shock, transfusion, pancreatitis, pneumonia, trauma, aspiration, or high-risk surgery; (2) comparison intervention: with and without anti-platelet therapy; (3) one or more the following outcomes: newly developed ARDS/ALI, hospital or 30-day mortality. In the case of duplicate publication, we only included studies that were the most informative and complete.

### Data Extraction and Quality Assessment

Two investigators (W. L. J. and L. H.) independently screened the abstracts or titles of the studies from the electronic search to identify all potential eligible studies and extracted data. Discrepancies were resolved by consensus or a third reviewer (G. X. F.). The following data were recorded: first author, publication year, country, study design, sample size, outcomes of the studies, and definition of anti-platelet therapy. We pooled the short-term mortality (hospital or 30-day mortality) and new development of ARDS/ALI with the adjusted data. We also checked the supplementary files and contacted the authors for available information where necessary.

The study level risk of bias was evaluated using the nine-star Newcastle-Ottawa Scale (NOS) [17], which consists of three major aspects: selection, comparability, and outcome. And we judged studies with a score of nine or eight stars to be at low risk of bias, seven or six stars to be at medium risk, and below six to be at high risk of bias.

### Statistical Analysis

All statistical analyses were performed using STATA, version 12.0 (Stata Corporation, College Station, Texas). A summary odds ratio (OR) was obtained on base of the adjusted OR in all analyses involved in this report. The hazard ratio (HR) and risk ratio (RR) were regarded as the eligible OR directly because all provided effect sizes of similar magnitude [18]. To calculate the adjusted OR with the 95% CI, a fixed-effects model was taken when significant heterogeneity was not present (the Q statistic (*p*≥0.1), I^2^ <50%). Elsewise, a random-effects model was used. We likewise performed predefined subgroup analyses to study the effect of antiplatelet therapy in critically ill patients. To assess the effect of a single study on pooled estimate, a sensi-tivity analysis was used by omitting one study in turn. Funnel plots and Egger’s linear regression tests were taken to evaluate publication bias [19]. A *P* value less than 0.05 was considered to indicate statistical signi-ficance.

## Results

### Study Selection and Study Characteristics

**Fig 1:**
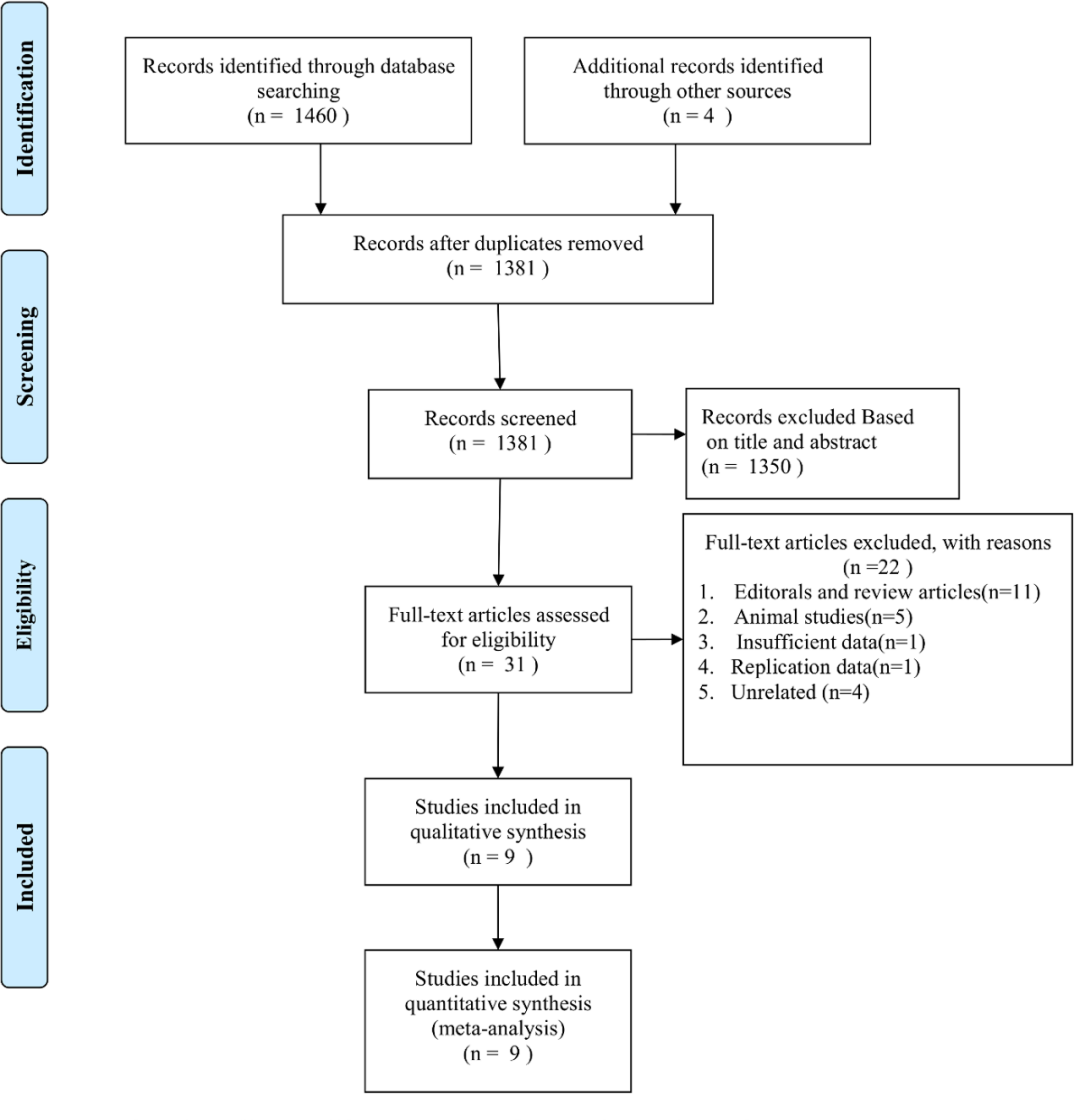
Flow diagram of study selection.

Fig 1 shows the detailed procedure of our literature search and selection. Our initial query yielded 1464 potentially relevant publications, of which 83 were excluded for duplicate studies and 1350 studies were excluded according to the titles and abstracts. After reviewing the full text of the remaining 31 studies, we identified 9 cohort studies in this present meta-analysis [14–15,20–26]. Of twenty-two full-text reports that were excluded, one [27] did not provide sufficient data to calculate the risk estimates. One report [28] was excluded because it was a duplicate study. Four [12,29–31] were excluded because they were unrelated. In addition, eleven reviews or editorial articles and five animal studies were likewise excluded.

The main characteristics of the nine selected cohort studies in this meta-analysis are shown in Table 1. In total, 14,612 patients (including 4765 cases of antiplatelet users) were enrolled in our meta-analysis. The main outcome of hospital or 30-day mortality was reported in six of nine articles [14–15,20–21,24,26]. Four of nine studies reported ARDS/ALI [15,22,23,25]. Four were pro-spective studies [15,22,24,26], and the residual were retrospective studies[14,20,21,23,25]. Only one study was population based. There was a congruous definition of ARDS/ALI between involved studies [32], but definitions of anti-platelet therapy included in the studies were not the same. The quality of the studies included was assessed by NOS scores, and the average NOS score of the involved studies was 6.9 (the range was 6 to 7). The detail is described in S1 File.

**Table 1:** Main characteristics of cohort studies included in this meta-analysis.

Study/Year	Country	No. total (Anti/Nonanti)	Study population	Study design	Main outcomes	Definition of antiplatelet therapy	Study quality*
Valerio-Rojas,2013 [14]	USA	651(272/379)	Adult ICU patients with severe sepsis and septic shock	SRC	Hospital mortality, ICU mortality, ARDS/ALI	Using ASA, clopidogrel, ticlopidine, dipyridamole at the time of ICU admission	7
Erlich, 2011[23]	USA	161(79/82)	Adult ICU patients with >1 predisposing ALI condition	PRC	ARDS/ALI, hospital mortality, ICU mortality	Using ASA, clopidogrel bisulfate, ticlopidine hydrochloride, cilostazol, dipyramidole, anagrelide, persantine at the time of hospital admission	7
Mazzeffi, 2015 [25]	USA	375(181/194)	Adult patients with aortic valve replacement surgery	SRC	ARDS	Taking aspirin within 5 days of surgery	7
Otto, 2013 [20]	Germany	886(187/699)	Adult ICU patients with severe sepsis or septic shock	SRC	Hospital mortality, ICU mortality	Taking ASA or clopidogrel during the ICU stay,a dose of 100mg/d ASA and 75mg/d clopidogrel or either of the anti-platelet drugs	7
Eisen, 2012 [21]	Australia	5523(2082/3441)	ICU patients with SIRS or sepsis	SRC	Hospital mortality, renal injury	Defined as ASA use for the 24-hr period around the time of SIRS recognition	7
Chalmers, 2008 [24]	UK	1007(311/696)	ED patients with community-acquired pneumonia	SPC	30-day mortality	Using aspirin on admission	6
Falcone, 2015 [26]	Italy	1005(390/615)	Adult ED patients with community-acquired pneumonia	SPC	30-day mortality	Using aspirin before and during hospitalization	7
Kor, 2011 [22]	USA	3855(976/2879)	Adult ED patients with >1 predisposing ARDS condition	MPC	ARDS, hospital mortality, ICU mortality	Defined as aspirin therapy pre-hospitalization	7
Chen, 2015 [15]	USA	1149(287/862)	Adult ICU patients with high risk for ARDS	SPC	ARDS/ALI, hospital mortality	Using aspirin before hospital admission	7

Anti/Nonanti antiplatelet/nonantiplatelet, ICU intensive care unit, ARDS acute respiratory distress syndrome, ALI acute lung injury, SPC single-centre prospective cohort, SRC single-centre retrospective cohort, MPC multi-centre prospective cohort, PRC population-based retrospective cohort, Study quality^*^ assessed by Newcastle-Ottawa Scale, ASA aspirin

### Meta-Analysis of Outcomes

**Fig 2:**
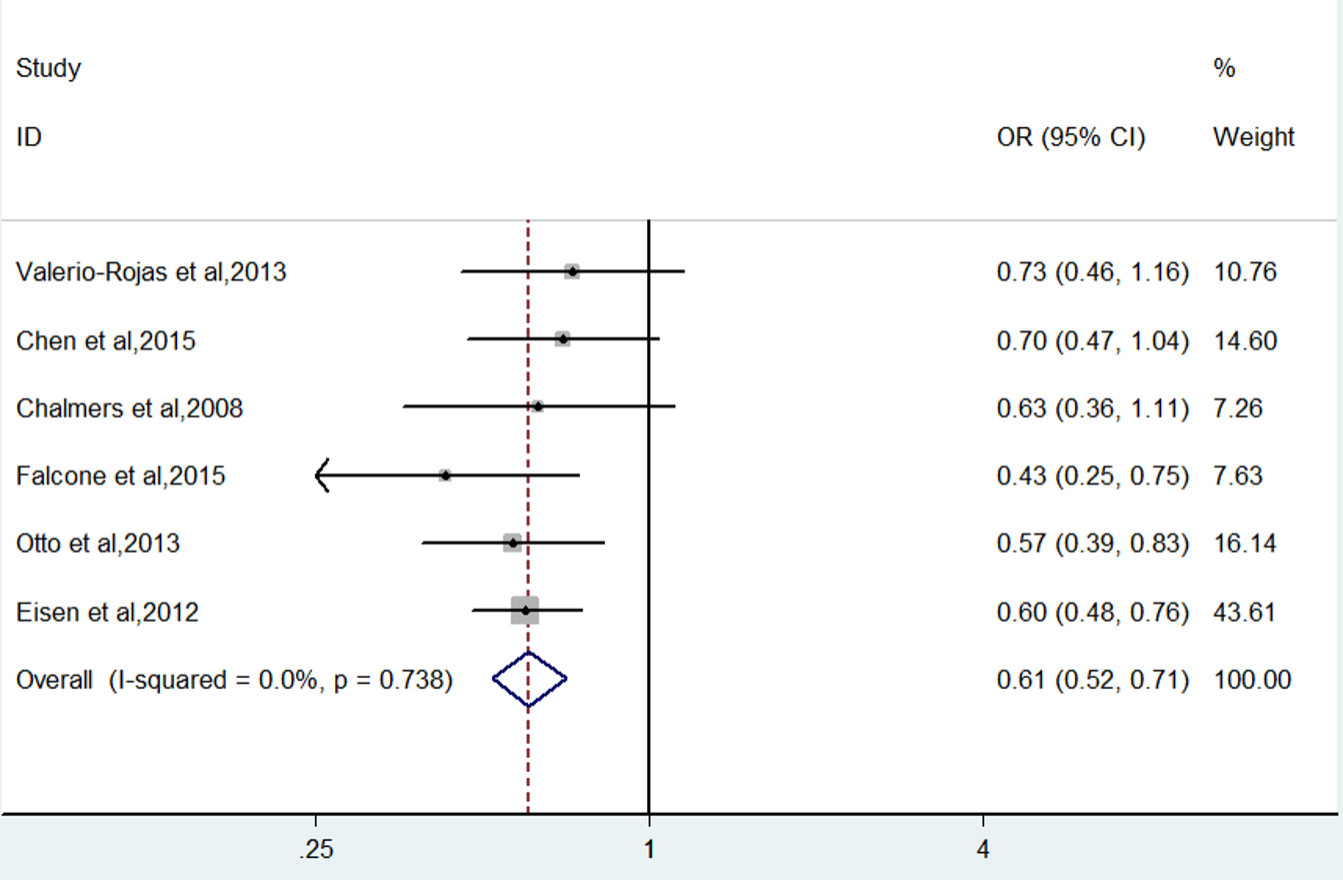
Forest plot showing the effect of anti-platelet therapy on mortality.

**Fig 3:**
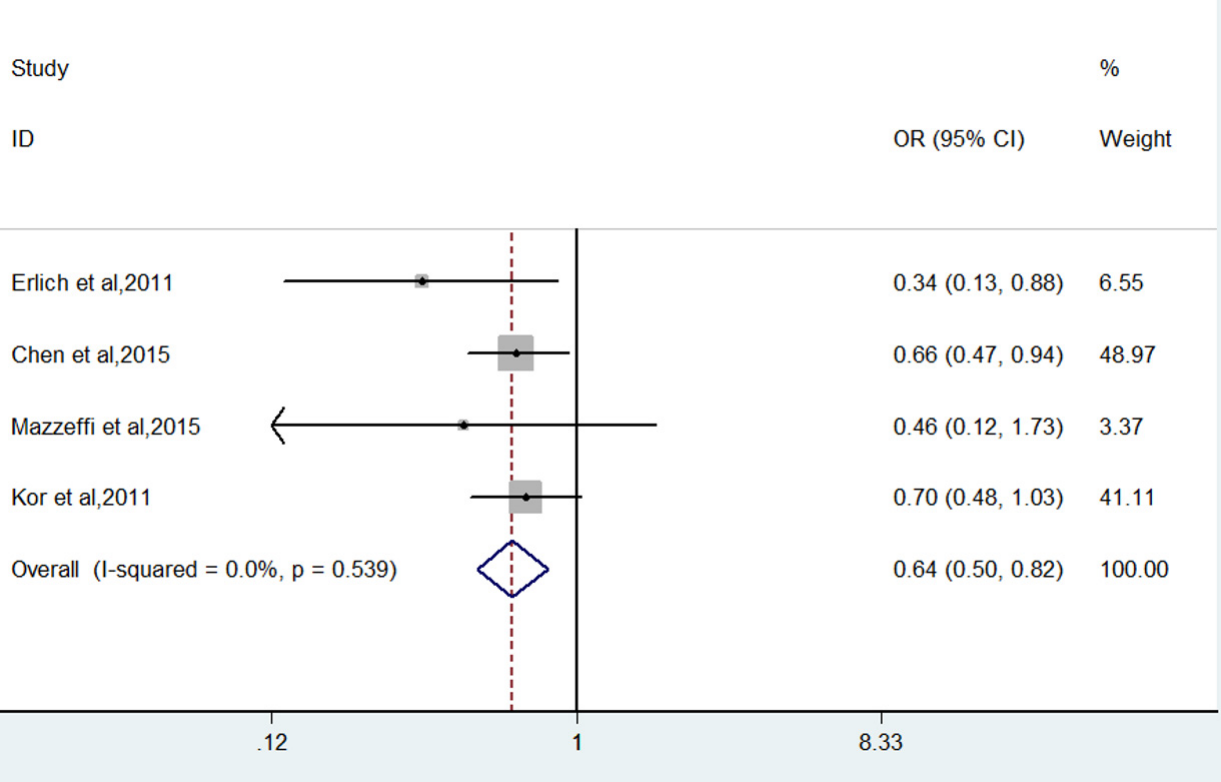
Forest plot showing the effect of anti-platelet therapy on newly developed ARDS/ALI.

Figs 2 and 3 show the pooled results from the fixed-effects model combining the adjusted ORs for hospital or 30-day mortality and ARDS/ALI, respectively. On base of a meta-analysis of with six reports, antiplatelet therapy was associated with a decreased risk of hospital or 30-day mortality (OR 0.61, (95% CI, 0.52 to 0.71); P <0. 001), with very low between-study heterogeneity (I^2^ = 0%; P = 0.738). We also carried out a meta-analysis of the incidence rate of newly developed ARDS/ALI with four studies. Similarly, the pooled results indicated that antiplatelet therapy had a beneficial impact on reducing ARDS/ALI (OR 0.64, (95% CI, 0.50 to 0.82); P <0. 001). There also was very low heterogeneity among the studies (I^2^ = 0%; P = 0.539).

### Subgroup and Sensitivity Analyses

To study the possibly existed heterogeneity, we performed a prespecified subgroup analysis according to predisposing conditions (sepsis/septic shock or > predisposing conditions), adjusted by propensity score (yes or no), type of mortality (hospital mortality or 30-day mortality), type of effect size (OR or HR), antiplatelet agents exposure (current exposure or former exposure), aspirin for antiplatelet therapy only (yes or no), and sample size (≤1000 or >1000). Our subgroup analyses suggested the effect of anti-platelet therapy on decreasing the incidence rate of mortality in ICU population with critical illness was consistent. Details can be shown in S2 File. In addition, sensitivity analyses suggested the overall combined OR didn^’^t materially alter by exclusion of any single study, with a range from 0.59 (95% CI, 0.50–0.70) to 0.63 (95% CI, 0.53–0.73).

### Publication Bias

**Fig 4:**
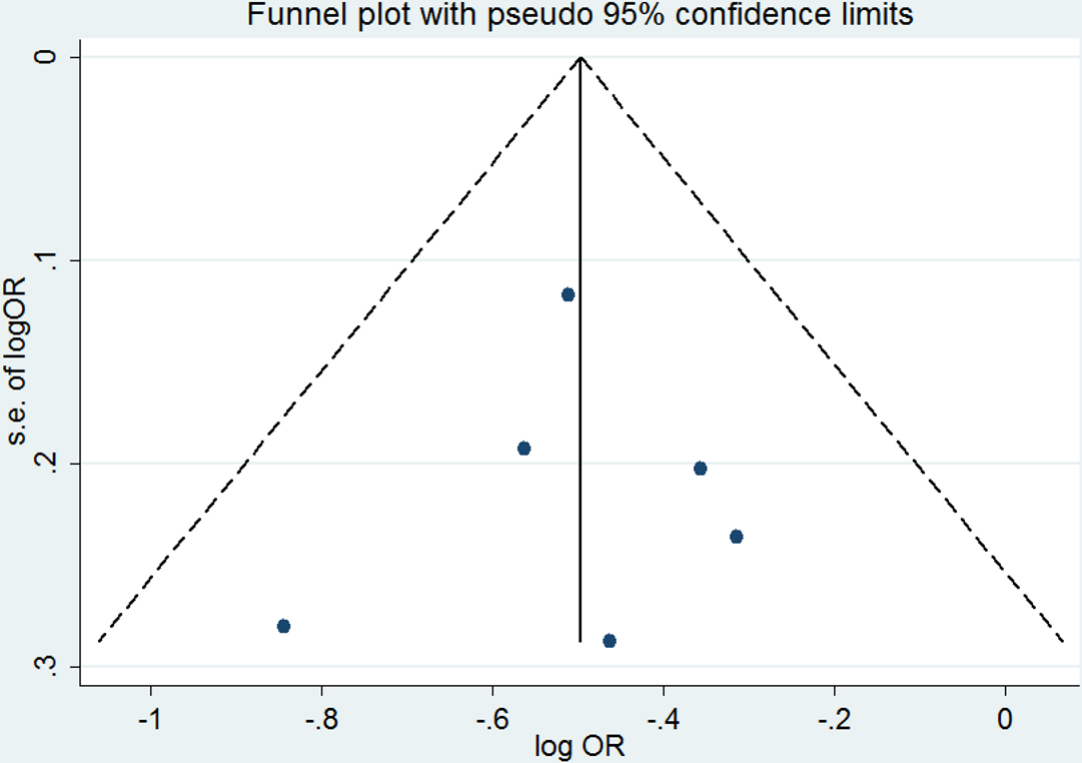
Funnel plot for the risk of mortality in critically ill patients.

The possibility of publication bias was judged by funnel plots and Egger^’^s test. There was no evidence of publication bias based on the funnels plot (Fig 4) and Egger^’^s test (mortality, *P* = 0.891; ARDS/ALI, *P* = 0.146). However, the low power with only six studies for mortality and four studies for ARDS/ALI confined the interpretability.

## Discussion

### Key Findings

The principal finding of our analysis is that antiplatelet therapy is associated with decrease in ARDS/ALI and mortality. To the best of our knowledge, this is the first meta-analysis to explore the effects of anti-platelet treatment on the risk of ARDS/ALI and mortality in critically ill patients with high-quality cohort study and adjusted data. Additionally, the beneficial effect of anti-platelet treatment on mortality was likewise present in every stratum from subgroup analyses. All this may suggest that the “plei-otropic effects” of anti-platelet therapy probably will be a novel therapeutic approach in critically ill patients in the future.

### Possible Mechanism

Although antiplatelet treatment may decrease the risk of mortality and ARDS/ALI in critically ill patients, the reason remains unclear and can be explained by several potential mechanisms. First, activated platelets may contribute to micro-vascular thrombosis and organ failure. Anti-platelet therapy can impede platelet activation and the surface expression of adhesion molecules, a pivotal process in mi-crovascular thrombus formation [33]. This viewpoint is proved by an animal study, in which platelet aggregation inhibitors abolished lipopolsaccharide (LPS)-induced thrombocytopenia and decreased fibrin deposition within the pulmonary microcirculation [34]. Nevertheless, the mortality rates in critically ill patients did not appear to be substantially influenced by heparin in two previously published meta-analyses [35–36]. Similarly, neither anti-thrombin III therapy nor tissue factor pathway inhibitor revealed a beneficial effect on survival [37–38]. Therefore, the anti-thrombosis function may be not the only possible mechanism of the benefit of anti-platelet treatment in critically ill patients. Second, antiplatelet agents also manifestly affect inflammatory processes and immune resposes. Platelets can modulate in-flammatory responses through: (1) releasing of pro- or anti-inflammatory compounds such as cytokines, chemokines; (2) releasing of antibacterial compounds and, together with neutrophils, trapping of bacteria; (3) mediating complement activation; (4) neutrophil-, macrophage- and endotheliocyte-mediated adhesion leading to alterations of cellular functions such as production of reactive oxygen species (ROS), cytokines, and chemokines as well as recruitment and immigration of leukocytes at the site of tissue damage[39]. Platelet activation also reveals a significant impact on the innate immune response through pathogen recognition by toll-like receptors (TLRs) and the expression of cell surface receptors [40]. And in an animal model of sepsis, anti-inflammatory agents have greatly shown a beneficial effect on mortality [41]. However, previous published randomized studies described a consistent result of no survival benefit of ibuprofen in sepsis [42–43]. Third, platelet activation also plays an important role in the pathobiology of disease processes. In the pathogenesis of ARDS, for instance, both an uncontrolled inflammatory response and dys-regulated coagulation are key pathways [44]. ARDS is largely predicated on the key roles for platelets in these two pathways. At sites of lung injury, platelets activation act as signalling molecules to propagate an immune response, promoting the recruitment of neutrophils to the damaged alveolus [45]. Besides, delayed neutrophil apoptosis is a remarkable feature of ARDS, allowing inflammatory cells to remain within the alveolar space, prolonging the period of lung injury and hypoxia [46]. An experimental study indicates that aspirin-triggered production of the anti-inflammatory lipid mediator 15-epi-lipoxin A4 restores neutrophil apoptosis and promotes the resolution of alveolar inflammation [47]. Further research is needed to determine whether the effect of anti-platelet treatment on improving patient outcomes is mediated via their microcirculatory improvement and/or anti-inflammatory effects, or others.

### Clinical Implications

Despite advancements into the pathophysiology of critically ill patients, no specific or reliable intervention has currently been developed [48–49]. Ascertaining a cost-effective and safe agent would represent a significant promotion in treating this deadly and costly condition, even if the benefit was low or adjuvant. Our findings suggested antiplatelet therapy might show a protective effect on decreasing the incidence rate of short-term mortality and ARDS/ALI. Accordingly, it may act as a prophylactic agent and/or as a treatment in critically ill patients, such as sepsis, ALI, and ARDS. Importantly, with a large number of the critically ill population, even a low rate of avoidable harm will be associated with massive preventable deaths. Consequently, it is imperative to identify the role of antiplatelet treatment as an adjunctive therapy in critically ill patients. In addition, in order to know the influence of exposure timing (former, current use) of antiplatelets on mortality in critically ill patients, we carried out a special subgroup analysis between current and former antiplatelet agent users. There were no obvious differences after comparison in these groups; however, current antiplatelet agent use revealed a stronger effect on survival benefits.

### Strengths and Weaknesses

The strength of this study is that there was an exhaustive search without language restrictions and PRISMA guidelines was used as our systematic review methods. Meanwhile, studies were assessed strictly for methodological quality, and only those of high quality were involved in our analysis. In addition, no significant heterogeneity between the included studies was observed in this study. However, our meta-analysis knows several limitations, the prin-cipal one being lack of randomized trials. Although more and more evidence emphasizes the potential benefit of antiplatelet therapy in critically ill patients, none of the randomized trials came out to report the relationship between the two described above. Considering the characteristics of observa-tional study, we should acknowledge selection bias and potential confounding. Actually, just like any observational study, it remains impossible to entirely exclude confounding, especially when the ob-served association is weak to some extent. Second, the definition of intensive care patients was wide and imprecise. In this study, critically ill patients was defined as the population with ALI/ARDS predisposing risk factors, involving sepsis, pancreatitis, transfusion, pneumonia, aspiration, trauma, or high-risk surgery. Nevertheless, it remains unclear whether the conclusion was fit for the unselected and board population with critical illness. Third, different definition of antiplatelet therapy in the included studies was used, and this may have a potential influence on our conclusion. Finally, unpub-lished studies or conference abstracts were not included. However, although publish bias was not found in our meta-analysis, unpublished studies or conference abstracts might be of great importance.

## Conclusions

In aggregate, our meta-analysis suggests that antiplatelet therapy is associated with decreased mortality, as well as a decreased incidence rate of ARDS/ALI in the critically ill, especially those with one or more predisposing risk factors such as high-risk surgery, trauma, pneumonia, and sepsis. However, it is also unknown whether similar results can be found in the unselected and broad population with critical illness. There is, thus, a great need for well de-signed, high-quality, large, ran-domized trials to confirm the effect of antiplatelet therapy in critically ill patients.

## Supporting Information

S1 File Quality assessment with Newcastle-Ottawa Scales. (DOC)

S2 File Subgroup analyses for mortality. (DOC)

S3 File PRISMA Checklist. (DOC)

S4 File Raw data for meta-analysis in our manuscript. (DOC)
