# Supplementary material for: Effect of Antiplatelet Therapy on Acute Respiratory Distress Syndrome and Mortality in Critically Ill Patients: A Meta-Analysis
Source: PLoS One. 2016 May 16;11(5):e0154754. doi: 10.1371/journal.pone.0154754 (PMC4868259; doi:10.1371/journal.pone.0154754)
Supplement: S1 File — (DOC) [file pone.0154754.s001.DOC]

| **Study** | **Selection** | | | | **Comparability** | **Outcome** | | | **Total score** |
| --- | --- | --- | --- | --- | --- | --- | --- | --- | --- |
|  | Exposed Cohort | Nonexposed Cohort | Ascertainment of exposure | Outcome of interest |  | Assessment of outcome | Length of follow-up | Adequacy of follow-up |  |
| Valerio-Rojas, 2013 [14] | * | * | * | * | ** | * | - | - | 7 |
| Erlich, 2011 [23] | * | * | * | * | ** | * | - | - | 7 |
| Mazzeffi, 2015 [25] | * | * | * | * | ** | * | - | - | 7 |
| Otto, 2013 [20] | * | * | * | * | ** | * | - | - | 7 |
| Eisen, 2012 [21] | * | * | * | * | ** | * | - | - | 7 |
| Chalmers, 2008 [23] | * | * | * | * | * | * | - | - | 6 |
| Falcone, 2013 [26] | * | * | * | * | ** | * | - | - | 7 |
| Kor, 2011 [22] | * | * | * | * | ** | * | - | - | 7 |
| Chen, 2015 [15] | * | * | * | * | ** | * | - | - | 7 |

**S1 File. Quality assessment with Newcastle-Ottawa Scales.**
