# Supplementary material for: Effect of Antiplatelet Therapy on Acute Respiratory Distress Syndrome and Mortality in Critically Ill Patients: A Meta-Analysis
Source: PLoS One. 2016 May 16;11(5):e0154754. doi: 10.1371/journal.pone.0154754 (PMC4868259; doi:10.1371/journal.pone.0154754)
Supplement: S2 File — (DOC) [file pone.0154754.s002.DOC]

**S2 File. Subgroup analyses for mortality.**

| **Subgroup** | **Studies, n** | **Reference** | **OR(95% CI), *P*** |
| --- | --- | --- | --- |
| Total | 9 | 14-15,20-21,24-26 | 0.61(0.52,0.71),<0.001 |
| Type of mortality | | | |
| hospital mortality | 4 | 14-15,20-21 | 0.63(0.53,0.74),<0.001 |
| 30-day mortality | 2 | 24,26 | 0.52(0.35, 0.77),0.001 |
| Type of effect size | | | |
| OR | 5 | 14-15,20-21,24 | 0.63(0.53, 0.73),<0.001 |
| HR | 1 | 26 | 0.43(0.25, 0.75),0.003 |
| Adjusted by propensity score | | | |
| Yes | 3 | 14-15,21 | 0.64(0.53, 0.77),<0.001 |
| No | 3 | 20,24,26 | 0.54(0.41, 0.71),<0.001 |
| Predisposing conditions | | | |
| Sepsis/septic shock | 3 | 14,20-21 | 0.61(0.51, 0.73),<0.001 |
| ＞1 predisposing conditions 3 | | 15,24,26 | 0.60(0.45, 0.80),<0.001 |
| aspirin for antiplatelet therapy only | | | |
| Yes | 2 | 14-15 | 0.58(0.48, 0.69),<0.001 |
| No | 4 | 20-21,24,26 | 0.71(0.53, 0.96),0.027 |
| Antiplatelets exposure | | | |
| current user | 4 | 14-15,24,26 | 0.63(0.50, 0.80),<0.001 |
| former user | 2 | 20-21 | 0.59(0.49, 0.72),<0.001 |
| Sample size | | | |
| ＜1,000 | 2 | 14,20 | 0.63(0.47,0.84),0.002 |
| ≥1,000 | 4 | 15,21,24,26 | 0.60(0.50,0.72),<0.001 |
