# Supplementary material for: Effect of Antiplatelet Therapy on Acute Respiratory Distress Syndrome and Mortality in Critically Ill Patients: A Meta-Analysis
Source: PLoS One. 2016 May 16;11(5):e0154754. doi: 10.1371/journal.pone.0154754 (PMC4868259; doi:10.1371/journal.pone.0154754)
Supplement: S4 File — (DOC) [file pone.0154754.s004.DOC]

**Table 1. OR (95%CI) on multivariate analysis for mortality**

| **Study/Year** | **OR** | **LCI** | **UCI** | **Endpoints** |
| --- | --- | --- | --- | --- |
| Valerio-Rojas et al,2013 | 0.73 | 0.46 | 1.16 | Hospital mortality |
| Chen et al,2015 | 0.7 | 0.47 | 1.04 | Hospital mortality |
| Chalmers et al,2008 | 0.63 | 0.36 | 1.11 | 30-day mortality |
| Falcone et al,2015 | 0.43 | 0.25 | 0.75 | 30-day mortality |
| Otto et al,2013 | 0.57 | 0.39 | 0.83 | Hospital mortality |
| Eisen et al,2012 | 0.6 | 0.48 | 0.76 | Hospital mortality |
| OR odd ratio, LCI lower confidential interval, UCI upper confidential interval. | | | | |

**Table 2. OR (95%CI) on multivariate analysis** for ARDS/ALI

| **Study/Year** | **OR** | **LCI** | **UCI** | **Endpoints** |
| --- | --- | --- | --- | --- |
| Chen et al,2015 | 0.659 | 0.469 | 0.944 | ARDS/ALI |
| Mazzeffi et al,2015 | 0.457 | 0.12 | 1.73 | ARDS |
| Kor et al,2011 | 0.7 | 0.48 | 1.03 | ALI |
| Erlich et al,2011 | 0.34 | 0.13 | 0.88 | ARDS/ALI |
| OR odd ratio, LCI lower confidential interval, UCI upper confidential interval, ARDS/ALI acute respiratory distress syndrome/acute lung injury. | | | | |
